# Gene selection and cancer type classification of diffuse large-B-cell lymphoma using a bivariate mixture model for two-species data

**DOI:** 10.1186/1479-7364-7-2

**Published:** 2013-01-05

**Authors:** Yuhua Su, Dahlia Nielsen, Lei Zhu, Kristy Richards, Steven Suter, Matthew Breen, Alison Motsinger-Reif, Jason Osborne

**Affiliations:** 1 Dr. Su’s Statistics, Department of Human Nutrition, Food, and Animal Sciences, University of Hawaii at Manoa, Honolulu, HI 96822, USA; 2 Bioinformatics Research Center, North Carolina State University, Raleigh, NC 27695, USA; 3 Biomarker and Predictive Analytics, GlaxoSmithKline, 5 Moore Drive, Research Triangle Park, NC 27709, USA; 4 Department of Genetic, University of North Carolina, Chapel HillUSA, NC 27599; 5 Department of Clinical Sciences, Center for Comparative Medicine & Translational Research, North Carolina State University, Raleigh, NC 27695, USA; 6 Department of Molecular Biomedical Sciences, College of Veterinary Medicine, North Carolina State University, Raleigh, NC 27695, USA; 7 Department of Statistics, North Carolina State University, Raleigh, NC 27695, USA

**Keywords:** Mixture models, Gene expression, Homology, Lymphoma

## Abstract

A bivariate mixture model utilizing information across two species was proposed to solve the fundamental problem of identifying differentially expressed genes in microarray experiments. The model utility was illustrated using a dog and human lymphoma data set prepared by a group of scientists in the College of Veterinary Medicine at North Carolina State University. A small number of genes were identified as being differentially expressed in both species and the human genes in this cluster serve as a good predictor for classifying diffuse large-B-cell lymphoma (DLBCL) patients into two subgroups, the germinal center B-cell-like diffuse large B-cell lymphoma and the activated B-cell-like diffuse large B-cell lymphoma. The number of human genes that were observed to be significantly differentially expressed (21) from the two-species analysis was very small compared to the number of human genes (190) identified with only one-species analysis (human data). The genes may be clinically relevant/important, as this small set achieved low misclassification rates of DLBCL subtypes. Additionally, the two subgroups defined by this cluster of human genes had significantly different survival functions, indicating that the stratification based on gene-expression profiling using the proposed mixture model provided improved insight into the clinical differences between the two cancer subtypes.

## Introduction

Diffuse large-B-cell lymphoma (DLBCL), the most common type of non-Hodgkin lymphoma in adults, accounts for 30% to 40% of newly diagnosed lymphomas and has an annual incidence in America of more than 25,000 cases. Combination chemotherapy has transformed DLBCL from a fatal disease into one that is often curable, but only approximately 50% of all patients are cured [1,2]. This suggests that DLBCL actually comprises several subgroups that differ in responsiveness to chemotherapy. The attempts to define subgroups of DLBCL have often failed due to diagnostic discrepancies. Clinically, the International Prognostic Index (IPI) [3] has been developed for use in the design of future therapeutic trials in patients with aggressive non-Hodgkin lymphoma and in the selection of appropriate therapeutic approaches for individual patients. However, IPI has not been used successfully to predict outcomes in DLBCLs so that patients can be stratified correctly for therapeutic trials. This may be attributed to the fact that the clinical factors of IPI (age, tumor stage, serum lactate dehydrogenase concentration, performance status, and number of extranodal disease sites) neither provide molecular insight into the heterogeneity of DLBCL nor identify specific therapeutic targets [4,5].

Recent developments in microarray technology allow researchers to accurately and precisely measure gene expression patterns in lymphomas which provides the opportunity to revolutionize the way these tumors are grouped and treated. In other words, studying gene expression profiles in lymphomas may provide the opportunity to identify pathways on which the tumor depends and to target the pathways for the development of new drugs. Indeed, gene-expression profiling studies have distinguished three molecular subtypes of DLBCL: germinal-center B-cell-like (GCB) DLBCL, activated B-cell-like (ABC) DLBCL, and primary mediastinal B-cell lymphoma (PMBL) [2,5-8].

The first attempt at examining gene expression profiling to identify distinct B-cell malignancies was made by Alizadeh et al. [9]. A hierarchical clustering algorithm [10] was used to group genes on the basis of similarity of their expression over all subjects. Subjects were also grouped based on the similarities in gene expression using the same clustering method. Two distinct subgroups of DLBCL were found based on the gene expression analysis: GCB DLBCL and ABC DLBCL. Alizadeh et al. [9] discovered that almost all genes that defined GCB DLBCL were highly expressed in normal germinal center B cells and, by contrast, most genes that defined ABC DLBCL were not expressed in normal germinal center B cells. In addition, there was a substantial and significant difference in the average five-year survival rate between patients with GCB DLBCL and ABC DLBCL.

Inspired by the work of Alizadeh [9], Rosenwald et al. [5] found that most of the genes with expression patterns that correlated with survival of the DLBCL subgroups fell within four gene-expression signatures. *A gene-expression signature is a group of genes expressed in a specific cell lineage or stage of differentiation or during a particular biologic response* and *hence genes within the same gene-expression signature are probably associated with similar biologic aspects of tumor*[5]. The authors in [5] then developed a molecular predictor consisting of 17 genes for the likelihood of survival after chemotherapy according to gene expression profiles of lymphomas. Shipp et al. [4] adopted the weighted-voting algorithm [11] to develop an outcome predictor with 13 genes and were able to classify two categories of DLBCL patients with very different five-year overall survival rates. Note that there is no overlap among the genes in the models derived in [4] and [5].

Wright et al. [8] formulated a DLBCL subgroup predictor based on Bayes’ rule, applied this method to the DLBCL gene expression data in [5], and constructed a 27-gene DLBCL subgroup predictor. Next, a new predictor including 14 genes among the previous predictor was constructed and applied to another set of gene expression data from DLBCLs [4]. Wright et al. [8] also demonstrated that the proposed algorithm can define cancer subgroups based on gene expression differences regardless of the DNA microarray platforms and could be used clinically to provide diagnostic information as the resulting survival rates were significantly different for the identified GCB and ABC DLBCL subgroups.

A panel of 36 genes whose expression predicts survival in DLBCL was identified by Lossos et al. [1] through literature review. They [1] selected 6 out of the 36 genes by ranking them on the basis of their predictive power for DLBCL survival obtained by univariate analysis. A 6-gene multivariate Cox proportional-hazards regression model for prediction of survival in DLBCL was constructed and applied to the data from [4] and [5]. Lossos et al. [1] concluded that the measurement of the expression of the six genes was sufficient to predict overall survival in DLBCL after stratifying patients into different risk groups based on their IPI score.

More recently, Blenk et al. [12] analyzed an enlarged data set (original data were generated by [5]) to confirm that there are clear expression differences between ABC and GCB DLBCL. To detect differentially expressed genes, they [12] used *limma* in [13] and further determined 50 best separating genes for class discovery. An optimal classifier with only 18 genes for distinguishing DLBCL subgroups was conducted. In addition, an optimal molecular survival predictor with only six genes was obtained. However, there was no overlap among the genes used in the classifier and the survival predictor established in [12].

Models introduced in [1,4,5,8,9,12] can be used to distinguish the subgroups in DLBCL and identify rational targets for research into treatment intervention. Moreover, the predictor identified by each study involved only a small number of genes and thus the needed DNA microarrays may be easily developed for clinical prediction. Nonetheless, genes seldom overlap in these models. Blenk et al. [12] showed that 6 of the 18 genes used in the optimal classifier were found again after analyzing another data set from [4]. However, none of these genes were identified in a subsequent investigation of survival [12].

Due to technical differences, the composition of the microarrays used, and the different algorithms used for constructing predictive models, it remains unclear which method and which model best captures the molecular and clinical heterogeneity of diffuse large-B-cell lymphoma. Therefore, the goal in this research was to give an example of how bivariate data can be used for clinical research.

## Methods

Let $X_{a_{\text{ij}}}$ and $X_{h_{\text{il}}}$ denote gene expression measurements from the *i*^th^ orthologous gene pair [14] for the *j*^th^ animal and the *l*^th^ human. The following independent linear models describe the association between gene expression and treatment (cancer type):

(1) $$\begin{array}{l} X_{a_{\text{ij}}}=\beta_{0a_{i}}+\beta_{1a_{i}}T_{a_{j}}+e_{a_{\text{ij}}}, \end{array}$$

(2) $$\begin{array}{l} X_{h_{\text{il}}}=\beta_{0h_{i}}+\beta_{1h_{i}}T_{h_{l}}+e_{h_{\text{il}}}, \end{array}$$

where $T_{a_{j}}$ and $T_{h_{l}}$ are {0,1} treatment indicators, and $e_{a_{\text{ij}}}$ and $e_{h_{\text{il}}}$ are independent $N(0,\sigma_{a}^{2})$ and $N(0,\sigma_{h}^{2})$ random variables. The $\sigma_{a}^{2}$ and $\sigma_{h}^{2}$ are variances for $e_{a_{\text{ij}}}$ and $e_{h_{\text{il}}}$, respectively. A given gene can be classified as non-differentially expressed (NDE, showing no signs of treatment effects), positively differentially expressed (pDE, showing positive treatment effects), or negatively differentially expressed (nDE, showing negative treatment effects). Furthermore, we assume dependency between differentially expressed orthologs. Therefore, for a human and animal gene pair, there are nine possibilities for categorizing this pair of genes, illustrated in Table 1.

**Table 1:** **Possible categories of**${\left(\beta_{1a_{i}},\beta_{1h_{i}}\right)}^{T}$

**Category**	$\left(\beta_{1a_{i}},\beta_{1h_{i}}\right)$	$\left(\mu_{\beta_{1a_{i}}},\mu_{\beta_{1h_{i}}}\right)$	$\text{Corr}(\beta_{1a_{i}},\beta_{1h_{i}})$
0	(NDE,NDE)	(0,0)	0
1	(pDE,pDE)	(+,+)	*ρ*_1_
2	(nDE,nDE)	(−,−)	*ρ*_2_
3	(pDE,nDE)	(+,−)	*ρ*_3_
4	(nDE,pDE)	(−,+)	*ρ*_4_
5	(NDE,pDE)	(0,+)	0
6	(NDE,nDE)	(0,−)	0
7	(pDE,NDE)	(+,0)	0
8	(nDE,NDE)	(−,0)	0

The following two-species nine-component bivariate normal mixture model in [15,16] is proposed to simultaneously model the vectors of the estimated treatment effects ${\left({\hat{\beta }}_{1a_{i}},{\hat{\beta }}_{1h_{i}}\right)}^{T}$ obtained from Equations 1 and 2:

(3) $$\begin{array}{lcrr} & \left(\begin{array}{c} {\hat{\beta }}_{1a_{i}}\\ {\hat{\beta }}_{1h_{i}} \end{array}\right)∼\Pi_{0}N\left(\begin{array}{cc} \left(\begin{array}{l} 0\\ 0 \end{array}\right), & \left(\begin{array}{cc} \sigma_{a0}^{2} & 0\\ 0 & \sigma_{h0}^{2} \end{array}\right) \end{array}\right) & & \\ & +\Pi_{1}N\left(\begin{array}{cc} \left(\begin{array}{c} \mu_{a1}\\ \mu_{h1} \end{array}\right), & \left(\begin{array}{cc} \sigma_{a1}^{2} & \rho_{1}\sigma_{a1}\sigma_{h1}\\ \rho_{1}\sigma_{a1}\sigma_{h1} & \sigma_{h1}^{2} \end{array}\right) \end{array}\right) & & \\ & +\Pi_{2}N\left(\begin{array}{cc} \left(\begin{array}{c} \mu_{a2}\\ \mu_{h2} \end{array}\right), & \left(\begin{array}{cc} \sigma_{a2}^{2} & \rho_{2}\sigma_{a2}\sigma_{h2}\\ \rho_{2}\sigma_{a2}\sigma_{h2} & \sigma_{h2}^{2} \end{array}\right) \end{array}\right) & & \\ & +\Pi_{3}N\left(\begin{array}{cc} \left(\begin{array}{c} \mu_{a3}\\ \mu_{h3} \end{array}\right), & \left(\begin{array}{cc} \sigma_{a3}^{2} & \rho_{3}\sigma_{a3}\sigma_{h3}\\ \rho_{3}\sigma_{a3}\sigma_{h3} & \sigma_{h3}^{2} \end{array}\right) \end{array}\right) & & \\ & +\Pi_{4}N\left(\begin{array}{cc} \left(\begin{array}{c} \mu_{a4}\\ \mu_{h4} \end{array}\right), & \left(\begin{array}{cc} \sigma_{a4}^{2} & \rho_{4}\sigma_{a4}\sigma_{h4}\\ \rho_{4}\sigma_{a4}\sigma_{h4} & \sigma_{h4}^{2} \end{array}\right) \end{array}\right) & & \\ & +\Pi_{5}N\left(\begin{array}{cc} \left(\begin{array}{c} 0\\ \mu_{h5} \end{array}\right), & \left(\begin{array}{cc} \sigma_{a5}^{2} & 0\\ 0 & \sigma_{h5}^{2} \end{array}\right) \end{array}\right) & & \\ & +\Pi_{6}N\left(\begin{array}{cc} \left(\begin{array}{c} 0\\ \mu_{h6} \end{array}\right), & \left(\begin{array}{cc} \sigma_{a6}^{2} & 0\\ 0 & \sigma_{h6}^{2} \end{array}\right) \end{array}\right) & & \\ & +\Pi_{7}N\left(\begin{array}{cc} \left(\begin{array}{c} \mu_{a7}\\ 0 \end{array}\right), & \left(\begin{array}{cc} \sigma_{a7}^{2} & 0\\ 0 & \sigma_{h7}^{2} \end{array}\right) \end{array}\right) & & \\ & +\Pi_{8}N\left(\begin{array}{cc} \left(\begin{array}{c} \mu_{a8}\\ 0 \end{array}\right), & \left(\begin{array}{cc} \sigma_{a8}^{2} & 0\\ 0 & \sigma_{h8}^{2} \end{array}\right) \end{array}\right), & & \end{array}$$

where *Π*_*k*_, *k *= 0,…,8 denote the mixing weights (the probability that an observation belongs to the *k*th component). Note that $\overset{8}{\underset{k=0}{\sum }}\Pi_{k}=1$ and *Π*_*k *_≥0. (*μ*_*ak*_,*μ*_*hk*_)^*T*^ and ${\left(\sigma_{\text{ak}}^{2},\sigma_{\text{hk}}^{2}\right)}^{T}$ are the vectors of the means and variances, respectively, for each species in each mixture component. *ρ*_*k*_ denotes the correlation between orthologs under the *k*th category. To accommodate the possible patterns of the gene expression for animal and human due to treatment intervention (different cancer types), the following constraints are imposed: *μ*_*a*1 _≥ 0, *μ*_*h*1 _≥ 0, *μ*_*a*2 _≤ 0, *μ*_*h*2 _≤ 0, *μ*_*a*3 _≥ 0, *μ*_*h*3 _≤ 0, *μ*_*a*4 _≤ 0, *μ*_*h*4 _≥ 0, *μ*_*h*5 _≥ 0, *μ*_*h*6 _≤ 0, *μ*_*a*7 _≥ 0, and *μ*_*a*8 _≤ 0; *ρ*_0 _= 0, *ρ*_5 _= 0, *ρ*_6 _= 0, *ρ*_7 _= 0, and *ρ*_8 _= 0.

Gene membership is determined according to the maximum posterior probability that an observation ${\left({\hat{\beta }}_{1a_{i}},{\hat{\beta }}_{1h_{i}}\right)}^{T}$ comes from the *k*^th^ component of the mixture.

A parametric bootstrap method in [17,18] to estimate the standard errors for the estimated parameters is provided. Bootstrapping is the practice of estimating properties of an estimator by measuring those properties when sampling from an approximating distribution. The basis of the bootstrap methodology is simple. In the parametric bootstrap setting, consider *F* to be a member of some prescribed parametric family and obtain ${\hat{F}}_{n}$ by estimating the family parameters, in this case, (*Π*_*k*_,*μ*_*ak*_,*μ*_*hk*_, Σ_*k*_)^*T*^, *k *= 0,…,8, from the data. In each iteration, by generating an *iid* random sequence, called a ‘resample’ from the distribution ${\hat{F}}_{n}$, new estimates of the parameters are obtained and the sampling properties (such as the mean, standard deviation, bias, and shape) can be evaluated.

The procedure of the parametric bootstrap resampling method to obtain the estimated standard errors of the estimated parameters for the nine-component mixture model is described as follows: 

1. ${\hat{F}}_{n}$ is formed by substituting the estimates of (*μ*_*ak*_,*μ*_*hk*_)^*T*^ and Σ_*k*_ into the 9-component mixture model (3).

2. The numbers of genes in category 0 through category 8 (*n*_0_,*n*_1_,…,*n*_8_) are drawn from a multinomial distribution with parameters *n* and **p**. *n* is the number of trials for each multinomial random variable. In this study, it is equal to the number of orthologs in two-species data. **p** is the vector of event probabilities for each trial. In this study, **p** is the vector of the mixing weights estimated from the data. The new mixing weights are then calculated for the bootstrap resampling and plugged into the nine-component mixture model (Equation 3) to form ${\hat{F}}_{n}$.

3. Bootstrap samples ${\left({\hat{\beta }}_{1a_{i}}^{*},{\hat{\beta }}_{1h_{i}}^{*}\right)}^{T}$ of size *n* are drawn from ${\hat{F}}_{n}$ formed above.

4. For each bootstrap resampling, obtain the numerically approximated maximum likelihood estimates for the parameters in the nine-component mixture using the expectation-maximization (EM) algorithm.

5. Repeat steps 1 to 4 *B* times independently. *B* is the number of bootstrap replications. Calculate the empirical standard deviation of a series of bootstrap replications of $\hat{\theta }$ accordingly. $\hat{\theta }$ is the estimator of *θ*, the parameter of interest. Since the standard error of the mean ( $s/\sqrt{n}$, sample standard deviation divided by the squared root of the size of the sample) is the estimate of the true standard deviation of the sample mean ( $\sigma /\sqrt{n}$, standard deviation for the population divided by the squared root of the size of the sample), essentially, the standard deviation of the bootstrap estimator obtained here is an estimation of the standard error of the mean for the parameter of interest. The bootstrap standard error ${\hat{\text{SE}}}_{B}$ of $\hat{\theta }$ is calculated as follows:

$$\hat{\text{SE}}{\left(\hat{\theta }\right)}_{B}=\sqrt{\frac{1}{B-1}\overset{B}{\underset{b=1}{\sum }}{\left({\hat{\theta }}_{b}^{*}-{\bar{\hat{\theta }}}^{*}\right)}^{2}},$$

where ${\hat{\theta }}_{b}^{*}$ is the estimator of *θ* calculated from the *b*^th^ bootstrap resample (*b *= 1,…,*B*), ${\bar{\hat{\theta }}}^{*}=\overset{B}{\underset{b=1}{\sum }}{\hat{\theta }}_{b}^{*}/B$; *B* is the total number of resamples (each of size *n*) collected from the empirical estimate of *F*.

### Data sources

In order to improve treatments for non-Hodgkin lymphoma in human and canine patients, researchers from North Carolina State University’s College of Veterinary Medicine and the University of North Carolina at Chapel Hill Lineberger Comprehensive Cancer Center conducted research to study tissue samples from human and canine non-Hodgkin lymphoma patients, with the hope of creating a genomic profile of non-Hodgkin lymphoma that would give oncologists and veterinarians greater insight into the disease’s biology and obtain the information that could lead to a clinical benefit for both species. The study protocol was approved by the Institutional Animal Care and Use Committee of North Carolina State University.

The team recruited dogs diagnosed with lymphoma to collect tissue samples for study. The dog data were measured at the probe set level on Affymetrix Canine Genome 2.0 array (Canine_2, Affymetrix Inc., Santa Clara, CA, USA), with a total number of probe sets equal to 43,035. Forty-eight dogs with one of the following diagnostic results were recruited: B-cell lymphoma (27 dogs), T-cell lymphoma (10 dogs), B-cell acute lymphoblastic leukemia (1 dog), T-cell acute lymphoblastic leukemia (4 dogs), and normal (6 dogs). Among the 27 dogs with B-cell lymphoma, 14 of them were diagnosed histopathologically with DLBCL. The 14 DLBCL patients could be further divided into two subgroups: 5 ABC DLBCL patients and 9 GCB DLBCL patients. For the purpose of this research, only data for the 14 dogs with DLBCLs were used. The dog microarray gene expression data were LOESS normalized by JMP Genomics 4.0 (Cary, NC, USA).

Corresponding data for human patients with lymphoma were extracted from the Gene Expression Omnibus (GEO) database [19]. Data for 460 lymphoma patients were retrieved from two series with GEO accession number: GSE10846 [7] and GSE11318 [6]. The human data were measured at the probe set level on Affymetrix Human Genome U133 2.0 array (HG-U133_Plus_2), with a total number of probe sets equal to 54,675. The human microarray gene expression data were also LOESS normalized by JMP Genomics 4.0. Based on the gene expression, two distinct subgroups were identified after principle component analysis. This implied that there may be a strong batch effect among the samples. Hence, only samples from one of these two subgroups were included in the data analysis. This resulted in 219 human subjects consisting of 31 PMBL, 78 ABC DLBCLs, 80 GCB DLBCLs, and 29 unclassified DLBCLs (distinguishing between subgroups of DLBCL is through gene-expression profiling [6,7]). To make the animal and human data comparable, only data for ABC and GCB DLBCLs with corresponding survival information were used. This resulted in a final dataset with 77 ABC DLBCL patients and 79 GCB DLBCL patients.

After averaging probe sets across a gene to obtain a gene-level transcript value, the orthologous information from HomoloGene release 64 at website ftp://ftp.ncbi.nih.gov/pub/HomoloGene/build64/ was applied to acquire the mappings between dog and human. This led to a total of 6,566 pairs of dog and human orthologs.

### Data analysis

We recall that the objective of the data analysis was to identify rational targets (genes) for treatment intervention simultaneously for both dog and human lymphoma patients with the hope of giving researchers greater insight into the disease’s biology. Furthermore, it was also of interest to verify if the targeted genes can serve as a good predictor to clinically distinguish subgroups of DLBCL in humans. Because the samples in humans that were used to estimate the distribution of the bivariate mixture model were also used to build the classification function, there was a possibility of over-fitting, resulting in a model that would indicate an over-optimistic separation between the subgroups than would be found in independent data. To avoid the biased classification result, a leave-one-out-cross-validation (LOOCV) procedure [20] was introduced as the following steps: 

1. Use all 14 observations for dogs and obtain the estimated coefficients of cancer type effect on gene expression ${\hat{\beta }}_{1a_{i}}$, *i *= 1,…,6,566. Omit one observation from the 156 human observations and obtain the estimated coefficients of cancer type effect ${\hat{\beta }}_{1h_{i}}$.

2. Use all ${\left({\hat{\beta }}_{1a_{i}},{\hat{\beta }}_{1h_{i}}\right)}^{T}$ to construct the nine-component bivariate normal mixture model. Identify gene membership accordingly.

3. Use genes classified into categories (1, 2, 3, and 4) (differentially expressed in both species) to develop a classification rule based on the remaining 155 human observations. Develop another classification rule based on genes classified into categories (1, 2, 3, 4, 5, and 6) (differentially expressed in human).

4. For the purpose of comparison, identify differentially expressed human genes by performing a single species analysis for human only. Choose genes based on the *p* values of the *t* statistics after adjusting for multiple comparison by controlling the false discovery rate (FDR) [21] at levels 0.01 and 0.00001.

5. Classify the holdout human observation using the classification rules constructed in steps 3 and 4.

6. Repeat steps 1, 2, 3, 4 and 5 until every one of the human observations is classified.

The classification rule was established through the *M*-dimensional centroid obtained from the *k*-means [22] clustering process applied to the training set. *M* was the number of genes retained for performing cancer type classification. *k* was equal to 2 as there were two types of cancer. SAS PROC FASTCLUS [23] was used to carry out the *k*-means clustering.

Since *k*-means does the clustering, and not the classification, the class of each cluster has to be determined for the classification rule before it can be used to classify future observations. Table 2 demonstrates the results of the *k*-means clustering.

**Table 2:** **The*k* -means clustering results**

	**Cluster 1**	**Cluster 2**
ABC DLBCL	*n*_11_	*n*_12_
GCB DLBCL	*n*_21_	*n*_22_

Clusters were designated so that the minimum misclassification rate was achieved. Therefore,

$$\;\left\{\;\;\begin{array}{l} \text{if}\;n_{12}+n_{21}<n_{22}+n_{11}\\ \Rightarrow \text{cluster 1 = “ABC DLBCL” and cluster 2 = “GCB DLBCL”},\\ \text{if}\;n_{12}+n_{21}>n_{22}+n_{11}\\ \Rightarrow \text{cluster 1 = “GCB DLBCL” and cluster 2 = “ABC DLBCL”},\\ \text{if}\;n_{12}+n_{21}=n_{11}+n_{22}\\ \Rightarrow \text{randomly assign cluster labels}. \end{array}\right)$$

The classification rule established at the *l*^th^ LOOCV procedure was then

$$\;\left\{\begin{array}{l} \text{if}\overset{M_{l}}{\underset{m=1}{\sum }}{\left(x_{h_{\text{ml}}}-\mu_{l,1m}^{\text{centroid}}\right)}^{2}<\overset{M_{l}}{\underset{m=1}{\sum }}{\left(x_{h_{\text{ml}}}-\mu_{l,2m}^{\text{centroid}}\right)}^{2}\\ \Rightarrow \text{classify the}\;l\text{th}\;\text{human subject into cluster 1,}\\ \text{else}\Rightarrow \text{classify the}\;l^{\text{th}}\;\text{human subject into cluster 2.} \end{array}\right)$$

*M*_*l*_ was the total number of genes retained at the *l*^th^ LOOCV procedure for cancer type classification. *l *= 1,…,156, as there were 156 human DLBCL patients. $x_{h_{\text{ml}}}$ was the *m*^th^ gene expression for the *l*^th^ hold-out human subject. For the *l*^th^ LOOCV procedure, $\mu_{l,1m}^{\text{centroid}}$ and $\mu_{l,2m}^{\text{centroid}}$ were the *m*^th^ centroid means (*m *= 1,…,*M*_*l*_) calculated from the *k*-means (*k *= 2) algorithm for cluster 1 and cluster 2, respectively.

## Results

### Parameter estimation

The maximum likelihood estimates of the parameters in the nine-component bivariate normal mixture model computed using the EM algorithm [24] are given in Table 3. The estimated mixture weight for category 0 was ${\hat{\Pi }}_{0}=0.823$ indicating approximately 5,404 (6,566 × 0.823) pairs of uninteresting dog and human orthologs. (*μ*_*a*_*μ*_*h*_)^*T*^ denotes the mean vector of each mixture component. Most of the estimated mean vectors were slightly larger in categories 1 through 4 than those in categories 5 through 8. It appeared that the magnitude of the estimated difference of expression in genes related to lymphoma in both species tended to be larger than in genes where differential expression was exhibited in only one species.

**Table 3:** Summary of parameter estimates for the bivariate mixture model averaged over the 156 LOOCV outcomes

	**Parameter**					
**Category**	***Π***_***k***_	***μ***_***ak***_	***μ***_***hk***_	$\sigma_{\text{ak}}^{2}$	***ρ***_***k***_***σ***_***ak***_***σ***_***hk***_	$\sigma_{\text{hk}}^{2}$
0	0.823(0.077)	NE	NE	0.013(0.001)	NE	0.012(0.001)
1	0.001(0.005)	0.341(0.081)	0.022(0.130)	0.004(0.010)	0.001(0.017)	0.022(0.060)
2	0.001(0.001)	-0.602(0.243)	-0.831(0.193)	0.199(0.082)	-0.128(0.048)	0.089(0.036)
3	0.001(0.004)	0.495(0.195)	-0.564(0.244)	0.042(0.078)	-0.042(0.041)	0.119(0.074)
4	0.000(0.003)	-1.131(0.422)	0.758(0.305)	0.000(0.028)	0.000(0.015)	0.000(0.050)
5	0.020(0.008)	NE	0.492(0.121)	0.020(0.021)	NE	0.058(0.049)
6	0.011(0.004)	NE	-0.517(0.136)	0.034(0.093)	NE	0.040(0.044)
7	0.130(0.077)	0.331(0.038)	NE	0.018(0.009)	NE	0.025(0.001)
8	0.012(0.008)	-0.478(0.339)	NE	0.011(0.051)	NE	0.042(0.054)

Numbers in parentheses are the bootstrap standard errors; *B*=5,000 (the number of bootstrap replications); *NE* not estimated.

### Gene selection and cancer type classification

**Figure 1:**
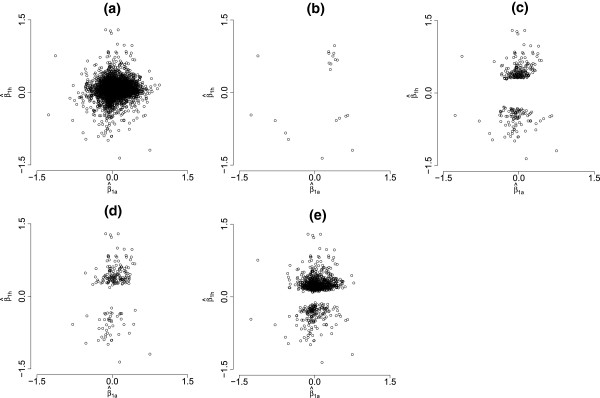
**Scatter plots of**${\left({\hat{\beta }}_{1a},{\hat{\beta }}_{1h}\right)}^{T}$**.** (**a**) all orthologs, (**b**) orthologs differentially expressed in both species (categories (1, 2, 3, and 4)), (**c**) orthologs for which the corresponding human genes are differentially expressed (categories (1, 2, 3, 4, 5, and 6)), (**d**) orthologs identified by analyzing the human data alone and controlling FDR at 0.00001, and (**e**) orthologs identified by analyzing the human data alone and controlling FDR at 0.01.

For the 156 LOOCV instances, the proposed mixture model determined 21 (14 genes appearing in the intersection of all hold-outs) human genes in categories (1, 2, 3, and 4) and 279 (185 genes appearing in the intersection of all hold-outs) human genes in categories (1, 2, 3, 4, 5, and 6). While analyzing the human data alone and controlling the FDR at levels 0.01 and 0.00001, 935 (706 genes appearing in the intersection of all hold-outs) and 190 (139 genes appearing in the intersection of all hold-outs) genes were identified as differentially expressed, respectively. Figure 1 shows the scatter plots of ${\left({\hat{\beta }}_{1a},{\hat{\beta }}_{1h}\right)}^{T}$ for all orthologs and the 21 pairs of orthologs in categories (1, 2, 3, and 4); i.e., the genes in this group showed evidence of distinguishing the two types of cancer for both species. Clearly, most of the pairs scattered around the (0,0) origin, indicating that these genes (for both species) did not have potential for serving as markers that could distinguish the two subgroups of DLBCL.

After identifying human genes that showed signs of discriminating between ABC DLBCL and GCB DLBCL and based either on two-species analysis (the nine-component bivariate mixture model) or on single-species (human only) analysis, the next step in the LOOCV procedure was to classify the hold-out human subject according to a classification rule established using the same set of genes. Table 4 summarizes the classification results over the 156 LOOCV instances under the four different gene selection criteria. It was interesting to see that using human genes from categories (1, 2, 3, and 4), categories (1, 2, 3, 4, 5, and 6) (two-species analysis), and the genes selected by controlling FDR at 0.00001 (single-species analysis) gave a very similar number of misclassifications, 17, 16, and 19, respectively. Nonetheless, choosing FDR = 0.01, the gene list was largely expanded. The enlarged gene list resulted in a very poor classification result: 79 subjects were misclassified, among whom the entire group of ABC DLBCLs were misclassified as GCB DLBCLs. Misclassification rates were 0.109, 0.103, 0.122, and 0.506, accordingly. It was reasonable to conclude that the classification results based on two-species (dog and human) data, in general, may outperform those based on only single-species (human) data.

**Table 4:** Misclassification tables using different criteria

	**Categories (1, 2, 3, and 4)**		**Categories (1, 2, 3, 4, 5, and 6)**		**FDR = 0.00001**		**FDR = 0.01**	
**Model prediction/subgroup**	**ABC**	**GCB**	**ABC**	**GCB**	**ABC**	**GCB**	**ABC**	**GCB**
ABC DLBCL	72	5	64	13	58	19	0	77
GCB DLBCL	12	67	3	76	0	79	2	77

### Prognostic DLBCL sub-categories defined by gene expression profiles

Does the taxonomy of DLBCL derived from gene expression patterns define clinically distinct subgroups of patients? To confirm that these two DLBCL subgroups defined by gene expression (the 21 genes in categories (1, 2, 3, and 4)) were both biologically and clinically distinct so that the mixture model approach could form the basis of a robust diagnostic test that may prove useful in assessing the results of therapeutic trials in DLBCL, overall survival and subgroup survival based on two types of gene-expression profiling, in [6] and [7] and the proposed mixture model approach, were plotted.

**Figure 2:**
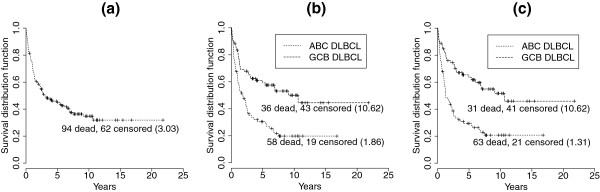
**Kaplan-Meier survival probability estimates for the dog and human lymphoma study.** (**a**) No stratification, (**b**) stratification based on the results of gene-expression profiling performed in [6] and [7], and (**c**) stratification based on the gene-expression profiling resulted from the proposed nine-component mixture model. Numbers in parentheses are medians.

Figure 2 shows the nonparametric Kaplan-Meier survival probability estimates [25] for patients with DLBCL under two situations, unstratified and stratified, by gene-expression profiling. Treating the DLBCL patients regardless of the biological difference between the subgroups gave a 5-year survival rate of 45%. The 5-year survival rates after stratifying the patients according to the gene-expression profiling performed in [6] and [7] were 31% for ABC DLBCL and 59% for GCB DLBCL as compared with the rates of 29% for ABC DLBCL and 64% for GCB DLBCL if patients were stratified by the gene-expression profiling results based on the proposed nine-component mixture model. Under both types of stratification, ABC and GCB DLBCL were associated with statistically significant differences in overall survival (*p* < 0.0001). A log-rank test [26] was used to test the hypothesis of equal survival functions. The molecular dissection of DLBCL by gene-expression profiling using the proposed nine-component mixture model apparently identified different features of these patients that influence their survival.

As determined by gene-expression profiling performed in [6] and [7], among the 156 patients, there were 77 ABC DLBCLs and 79 GCB DLBCLs. Conversely, the stratification stated by the gene-expression profiling using the proposed nine-component mixture model gave a result of 84 ABC DLBCLs and 72 GCB DLBCLs. More specifically, five of the ABC DLBCLs had been classified as GCB DLBCLs, and 12 of the GCB DLBCLs had been categorized as ABC DLBCLs. However, the difference between the median survival time (years) of the subgroups stratified by gene-expression profiling performed in [6] and [7] was smaller than that of the subgroups stratified by gene expression profiling using the proposed nine-component mixture model (8.76 vs. 9.31). This may imply that the stratification based on the gene expression profiling using the proposed nine-component mixture model provided better insight for the clinical difference between ABC and GCB DLBCL. These results suggested that the microarray-based outcome predictor not only reflected the clinical difference between the two DLBCL subgroups, but also provided a possible strategy of investigation for further individualization of patient treatment.

### Justification of the 21 selected human genes

To validate the relevance between specific genes and phenotypes, a careful search of the literature was undertaken using Entrez Gene [27]. Some of these 21 genes (the genes in Table 5 with Entrez ID highlighted in italics) were identified by this search as potentially associated with the development of DLBCL. A brief summary of the relationship between these candidate genes and the development of DLBCL is given as follows: 

*CD39* [Entrez Gene:953] is a B lymphocyte activation marker and has powerful functions in the immune system [28].

**Table 5:** Summary of the gene-specific information (retrieved from Entrez Gene, an NCBI’s database for gene-specific information)

**Entrez Gene ID**	**Category**	**Dog ortholog**	**Frequency**^**a**^	**Symbol**	**Official full name**
*953*	1	486810	156	CD39; ATPDase;	Ectonucleoside triphosphate
				FLJ40921; FLJ40959;	diphosphohydrolase 1
				NTPDase-1; DKFZp686D194; DKFZp686I093; ENTPD1	
1278	2	403824	156	OI4; COL1A2	Collagen, type I, alpha 2
2530	1	448804	156	MGC26465; FUT8	Fucosyltransferase 8 (alpha (1,6) fucosyltransferase)
*4005*	2	609006	156	TTG2; RBTN2; RHOM2;	LIM domain only 2 (rhombotin-like 1)
				RBTNL1; LMO2	
*4033*	3	486631	156	JAW1; LRMP	Lymphoid-restricted membrane protein
5919	2	475532	156	TIG2; HP10433; RARRES2	Retinoic acid receptor responder (tazarotene induced) 2
*6003*	3	612789	124	MGC17173; RGS13	Regulator of G-protein signaling 13
6856	3	475889	154	SYPL; H-SP1; SYPL1	Synaptophysin-like 1
6925	1	403949	156	E2-2; ITF2; PTHS; SEF2;	Transcription factor 4
				SEF2-1; SEF2-1A; SEF2-1B;	
				bHLHb19; MGC149723;	
				MGC149724; TCF4	
7037	1	403703	11	TFR; CD71; TFR1; TRFR;	Transferrin receptor (p90, CD71)
				TFRC	
9435	1	485701	156	C6ST; GST2; GST-2; Gn6ST-1; CHST2	Carbohydrate (*N*-acetylglucosamine-6-*O*) sulfotransferase 2
9760	2	486964	156	TOX1; KIAA0808; TOX	Thymocyte selection-associated high mobility group box
10447	3	612336	154	ILEI; GS3786; FAM3C	Family with sequence similarity 3, member C
23075	3	485385	4	HSPC321; SWAP-70;	SWAP switching B-cell complex 70kDa
				FLJ39540; KIAA0640;	subunit
				SWAP70	
25816	1	481428	156	GG2-1; SCCS2; SCC-S2;	Tumor necrosis factor, alpha-induced
				MDC-3.13; TNFAIP8	protein 8
*27086*	1	484692	156	QRF1; 12CC4; hFKH1B;	Forkhead box P1
				HSPC215; FLJ23741;	
				MGC12942; MGC88572;	
				MGC99551; FOXP1	
56941	2	484628	156	DC12; MGC111075;	Chromosome 3 open reading frame 37
				C3orf37	
81552	1	608562	145	ECOP; GASP; FLJ20532;	Vesicular, overexpressed in cancer,
				DKFZp564K0822; VOPP1	prosurvival protein 1
81641	1	479002	116	Apm; Apn; KZP; AP-M; AP-N; Lap1; rAPN; Anpep	Alanyl (membrane) aminopeptidase
121355	4	477590	156	FAM112B; FLJ32942;	Gametocyte specific factor 1
				GTSF1	
219972	1	475960	156	MPG1; MGC132657;	Macrophage expressed 1
				MGC138435; MPEG1	

For the 21 human genes in categories (1, 2, 3, and 4) determined by the bivariate mixture model.

Genes in *italics* were identified by this search as potentially associated with the development of DLBCL.

^a^number of times the corresponding gene was found in the classification gene set for the 156 LOOCV instances.

The expression pattern of *JAW1* [Entrez Gene:4033], a lymphoid-restricted protein, suggested that this protein may have a role in the developmentally regulated trafficking of the antigen receptors in B cells and may influence lymphoid development [29]. Tedoldi et al. [30] pointed out that high levels of *Jaw1* mRNA were found in germinal center B-cells and in diffuse large B-cell lymphomas of germinal center subtype.

The importance of *LMO2* [Entrez Gene:4005], though its function in germinal center cells is unknown, as a candidate marker involved in the development of DLBCL has been discussed in several papers. Natkunam et al. [31] studied *LMO2* at the protein level and confirmed that *LMO2* is expressed specifically in germinal center B cells, which is fully in keeping with gene-expression profiling studies that showed high levels of *LMO2* mRNA in germinal center B cells. They in [31] also observed that among DLBCLs, *LMO2* tended to be expressed in cases assigned by phenotyping to the GCB categories and can therefore be added to the panel of markers that pathologists may use to subcategorize lymphomas. Morton et al. [32] claimed that *LMO2* is one of the candidate genes involved in lymphocyte development and is highly expressed in germinal center lymphocytes. Durnick et al. [33] studied the relationship between *LMO2* expression and *t(14;18)/IGH-BCL2*, a specific marker of lymphomas of germinal center origin and has been specifically associated with the GCB subgroup of DLBCL as determined by gene expression profiling but not in the ABC cases. There was a statistically significant association between *IGH-BCL2* fusion and *LMO2* protein expression and hence *LMO2* was suggested as a potential marker for the GCB phenotype. A similar conclusion has also been reached by [1].

Germinal center B lymphocytes prominently express at least two regulators of G-protein signaling (RGS) proteins, *RGS1* and *RGS13* [Entrez Gene:6003]. RGS is a family of proteins acting to limit and modulate heterotrimeric G-protein signaling. Han et al. [34] discovered that RGS1 and RGS13 act together to regulate chemokine receptor signaling in human germinal center B lymphocytes. The results provide some insight toward finding methods to reduce or eliminate an organism’s negative reaction to a treatment stimulus.

The importance of the transcription factor *FOXP1* [Entrez Gene:27086] as marker for the activated B-cell-like signature has been well-established [5,9]. Banham et al. [35] investigated the prognostic importance of FOXP1 protein expression in DLBCL and found that the overall empirical survival curves for the two subgroups based on the expression of *FOXP1* are significantly different. Goatly et al. [36] made an attempt to discover the underlying molecular mechanism of *FOXP1* expression in lymphoma development by investigating the *FOXP1* translocation, copy number change, and protein expression in mucosa-associated lymphoid tissue lymphoma and DLBCL. Korac and Dominis [37] explored the association between *FOXP1*, *BCL2*, and *BCL6* gene expression in diffuse large B-cell lymphoma tumor cells. *FOXP1* protein was detected in 28 patients; genetic abnormalities involving the *FOXP1* locus were found in 19 patients, and both were present in 13 patients, among the samples of lymph nodes from 53 patients with newly diagnosed DLBCL. *FOXP1* genetic abnormalities have been found to be associated with both *BCL2* and *BCL6* expression. Though it has been discovered that *BCL2* and *BCL6* proteins have an impact on diffuse large B-cell lymphoma development and outcome, they may not be good prognostic markers. *FOXP1* has played a role in the development of DLBCL. The identified association among *FOXP1*, *BCL2*, and *BCL6* indicates the possibilities of uncovering the development process in diffuse large B-cell lymphoma tumor cells. In addition, Nyman et al. [38] used *FOXP1* and *MUM1/IRF4* as activated B-cell-like markers to distinguish patients between the activated B-cell-like and other diffuse large B-cell lymphoma subtypes. Most recently, six common prognostic biomarkers, including *FOXP1*, were used to conclusively decide the cut-off values calculated by receiver operating curves to predict survival for DLBCL patients [39]. All these results suggested that FOXP1 expression may be important in DLBCL pathogenesis.

Among the 21 selected genes, five (from categories 1, 2, and 3) have been carefully examined to explore their association with the development of lymphoma. From the mixture model assumption, genes in the same category should react to the stimulus (drug treatment, cancer type, etc.) in a similar manner. Hence, the implications of these 21 genes (some of them may not have been studied scrupulously) in lymphoma may provide timely and important insight on guiding future investigations of their roles in both B-cell biology and lymphoma development.

## Conclusions

Since the development of high throughput gene expression technology, the important and difficult task of searching for genes that exhibit differences across species (cancer types or treatment groups in drug trials) has been the focus of much research. Simultaneously analyzing gene expression across two species takes into account the biological similarity between different organisms while identifying genes that could be potential prognostic markers and increase the power to detect differences. Identification of the relevant genes and a better understanding of the associated molecular pathways may open new possibilities in cancer diagnosis and treatment. Furthermore, it may become a practical assay for newly diagnosed patients to optimize their clinical management.

In this case study, the application of the proposed nine-component mixture model successfully reduced the quantities of variables (genes) needed to be investigated for the study of two types of DLBCL in humans. The dimension of variables decreased from 6,566 to 21, a cluster of genes that were identified as being differentially expressed in both species. On the other hand, an analysis of data from one species that selected genes using a specified FDR led to a much longer list of differentially expressed human genes (935 genes with FDR = 0.01 and 190 with FDR = 0.00001). Furthermore, the misclassification rate for human cancer type classification using clustering with gene expression from these 21 genes identified by the bivariate mixture model was remarkably low. The survivorship of the patients stratified according to this clustering was very different across the two types of cancer, indicating that the stratification based on gene-expression profiling using the proposed nine-component mixture model provided better insight for the clinical differences between the two types of cancer.

While validating the relevance of the identified human genes through NCBI’s database, literature, if any, for the corresponding dog orthologs were also searched. Far less research about DLBCL has been conducted for canines. As the model assumption is based on the biological mechanism behind humans and animals, the promising DLBCL classification results based on the human genes may be extended to dogs. Furthermore, currently, direct experiments on humans are not practical. This research provides the possibility for scientists to conduct observational or experimental research on modeling organisms as the first step to understand phenotypes, and then extend the findings to humans for further investigation.

## Abbreviations

DLBCL: diffuse large-B-cell lymphoma; GCB DLBCL: germinal-center B-cell-like diffuse large-B-cell lymphoma; ABC DLBCL: activated B-cell-like diffuse large-B-cell lymphoma.

## Competing interests

The authors declare that they have no competing interests.

## Authors’ contributions

All authors read and approved the final manuscript.
